# Effects of PI and PIII Snake Venom Haemorrhagic Metalloproteinases on the Microvasculature: A Confocal Microscopy Study on the Mouse Cremaster Muscle

**DOI:** 10.1371/journal.pone.0168643

**Published:** 2016-12-16

**Authors:** Cristina Herrera, Mathieu-Benoit Voisin, Teresa Escalante, Alexandra Rucavado, Sussan Nourshargh, José María Gutiérrez

**Affiliations:** 1 Facultad de Farmacia, Universidad de Costa Rica, San José, Costa Rica; 2 Instituto Clodomiro Picado, Facultad de Microbiología, Universidad de Costa Rica, San José, Costa Rica; 3 William Harvey Research Institute, Barts and The London School of Medicine and Dentistry, Queen Mary University of London, London, United Kingdom; Instituto Butantan, BRAZIL

## Abstract

The precise mechanisms by which Snake Venom Metalloproteinases (SVMPs) disrupt the microvasculature and cause haemorrhage have not been completely elucidated, and novel *in vivo* models are needed. In the present study, we compared the effects induced by BaP1, a PI SVMP isolated from *Bothrops asper* venom, and CsH1, a PIII SVMP from *Crotalus simus* venom, on cremaster muscle microvasculature by topical application of the toxins on isolated tissue (i.e., *ex vivo* model), and by intra-scrotal administration of the toxins (i.e., *in vivo* model). The whole tissue was fixed and immunostained to visualize the three components of blood vessels by confocal microscopy. In the *ex vivo* model, BaP1 was able to degrade type IV collagen and laminin from the BM of microvessels. Moreover, both SVMPs degraded type IV collagen from the BM in capillaries to a higher extent than in PCV and arterioles. CsH1 had a stronger effect on type IV collagen than BaP1. In the *in vivo* model, the effect of BaP1 on type IV collagen was widespread to the BM of arterioles and PCV. On the other hand, BaP1 was able to disrupt the endothelial barrier in PCV and to increase vascular permeability. Moreover, this toxin increased the size of gaps between pericytes in PCV and created new gaps between smooth muscle cells in arterioles in *ex vivo* conditions. These effects were not observed in the case of CsH1. In conclusion, our findings demonstrate that both SVMPs degrade type IV collagen from the BM in capillaries *in vivo*. Moreover, while the action of CsH1 is more directed to the BM of microvessels, the effects of BaP1 are widespread to other microvascular components. This study provides new insights in the mechanism of haemorrhage and other pathological effects induced by these toxins.

## Introduction

Viperid snakebite envenomings are characterized by drastic alterations in the microvasculature, which cause local and systemic haemorrhage and alterations in tissue regenerative processes [1,2]. Zinc-dependent snake venom metalloproteinases (SVMPs) are largely responsible for these effects, being abundant components in viperid snake venoms [3]. In addition, they are also involved in the pathogenesis of other aspects of local tissue alterations, such as myonecrosis, blister formation, inflammation, and oedema [4].

SVMPs have been classified in three groups according to their domain structure: 1) PI, which comprise only the metalloproteinase domain; 2) PII, which have, in addition to catalytic domain, a disintegrin domain; and 3) PIII, which present a catalytic domain followed by a disintegrin-like domain and a cysteine-rich domain [5]. In general, PIII SVMPs have higher haemorrhagic activity than PI SVMPs. Moreover, differences in the tissue distribution between haemorrhagic PI and PIII SVMPs have been described [6,7], which could have implications in their capacity to induce microvascular damage and haemorrhage.

It has been proposed that the mechanism by which SVMPs disrupt the microvasculature is by hydrolyzing basement membrane (BM) and other extracellular matrix components, thus causing weakening of the mechanical stability of capillaries, and subsequent loss of endothelial cells integrity and extravasation of blood components due to the action of hemodynamic biophysical forces operating in the microvasculature [8,9]. However, the precise mechanism by which SVMPs disrupt the microvasculature, and whether there are differences in the actions between the different types of SVMPs that could explain variations in their haemorrhagic activity, have not been completely elucidated. Moreover, it has been described that extravasation in venules due to the prominent inflammatory reaction characteristic of these envenomings may also contribute to the haemorrhagic mechanism [10–12]. There is very little information on the action of haemorrhagic SVMPs on the various types of vessels in the microvasculature, i.e. capillaries, venules and arterioles.

Several studies have demonstrated the ability of haemorrhagic SVMPs to hydrolyze proteins of the BM and other extracellular components *in vitro* [7,13–17]. In contrast, *in vivo* studies on the action of these toxins are scarce. Previous investigations have used three methodological approaches for assessing the action of SVMPs *in vivo*: immunohistochemistry in tissue sections, immunodetection by Western blot in tissue homogenates or in exudates, and proteomics analysis of wound exudates collected in the vicinity of affected tissue [6,7,16–18]. Despite the relevant contribution of these studies to the understanding of the mechanism(s) of action of SVMPs, in most of them the nature of BM components and their fragments is unknown. Moreover, these methodologies have not allowed a differential analysis of SVMPs effects on the various blood vessel types in the whole tissue. Therefore, there is a need for novel *in vivo* models to study the effects of SVMSs on the different components of the microvasculature using a more detailed and quantitative approach that could complement previous investigations and provide a more comprehensive picture of this relevant pathology.

Due to the transparency and thinness of the cremaster muscle, it is a highly convenient tissue to analyze histological changes by light microscopy. Previous studies have used this muscle to investigate the effects of snake venoms and isolated toxins on the microvasculature by intravital microscopy with low resolution techniques, which allow the observation of venom- and SVMP- induced haemorrhage [19–21]. The use of cremaster muscle for confocal microscopy allows the collection of high-resolution images in three dimensions of longitudinal blood vessels in whole tissue preparations, thus enabling a more detailed and quantitative analysis of microvascular components.

In the present study, we compared the effects of two haemorrhagic SVMPs: BaP1, a PI from *Bothrops asper* venom, considered a weak haemorrhagic toxin; and CsH1, a PIII from *Crotalus simus* venom that has a higher haemorrhagic activity. The action of these SVMPs was studied on the three components of blood vessels, i.e. BM, endothelial cells, and smooth muscle cells/pericytes of the cremaster muscle microvasculature, using an immunofluorescence approach by confocal microscopy. In addition, the role of blood flow and the differential effects of SVMPs on the three vessel types: capillaries, venules and arterioles, were studied. Our findings demonstrate differences in the ability of both SVMPs to degrade type IV collagen in the presence or absence of blood flow, and between the different vessel types. Moreover, differences were observed in the action of these SVMPs on endothelial cell-cell junctions, and on smooth muscle cells and pericytes. This study provides new insights in the mechanism of action of haemorrhagic SVPMs, and describes for the first time novel effects of SVMPs to various components of the microvasculature.

## Materials and Methods

### Isolation of SVMPs

The PI SVMP BaP1 was isolated from the venom of *Bothrops asper*, as described by Gutiérrez et al. [22] and Watanabe et al. [23], by a combination of ion-exchange chromatography on CM-Sepharose, followed by affinity chromatography on Affi-gel Blue. The PIII SVMP CsH1 was isolated from *Crotalus simus* venom, as described by Herrera et al. [7], by ion-exchange chromatography on DEAE-Sepharose, followed by gel filtration on a Superdex TM 200 10/300GL (GE Healthcare, LifeSciences) column (10 x 300 mm) using an ÄKTA FPLC (GE Healthcare, Life Sciences). Homogeneity of SVMP preparations was assessed by SDS-polyacrylamide gel electrophoresis (SDS-PAGE). Both toxins were isolated from the venoms of more than 40 adult specimens of each species collected in Costa Rica and maintained at the serpentarium of Instituto Clodomiro Picado, Costa Rica. After collection, venoms of each species were separately pooled, lyophilized, and stored at -20°C until used. The Minimum Hemorrhagic Dose (MHD), corresponding to the amount of enzyme that induces a hemorrhagic spot of 10 mm diameter in mice 2 h after injection, is 20 μg for BaP1 [22] and 2.2 μg for CsH1 [7].

### Ethics statement

Inbred male C57BL/6 mice (20–25 g body weight) were purchased from Charles River Laboratories, Cambridge, UK and Laboratorio de Ensayos Biológicos, LEBI, Costa Rica. The protocols involving the use of animals were approved by the Animal Welfare and Ethical Review Board (AWERB), Queen Mary University of London, and the Institutional Committee for the Care and Use of Laboratory Animals (CICUA), University of Costa Rica, and meet the International Guiding Principles for Biomedical Research Involving Animals (CIOMS) and UK legislation for the protection of animals. Mice were maintained under standard conditions of temperature (22±2°C), light/dark cycles of 12 h, and food and water *ad libitum*.

### *Ex vivo* effects of SVMPs on cremaster muscle vasculature (model without blood flow)

Groups of four mice were sacrificed by cervical dislocation and the cremaster muscle was dissected out. The isolated muscles were incubated, at room temperature, with either BaP1 or CsH1 SVMPs dissolved in 100 μL of 0.12 M NaCl, 0.04 M phosphate, pH 7.2 solution (PBS). Initially, three doses of BaP1 (10, 30 and 100 μg) and two incubation times (5 and 15 min) were evaluated in order to study the dose and time dependence of the effects. The dose of 30 μg of BaP1 and the incubation time of 15 min were selected for further studies. In the case of CsH1, a dose of 15 μg was selected as to induce a haemorrhage of similar intensity in the cremaster muscle to that caused by 30 μg of BaP1 by intravital microscopy, as described previously [21]. This 2:1 mass ratio of the SVMPs corresponds to an approximate molar ratio of 5:1. Control tissues were incubated with PBS alone. After incubation, tissues were washed three times with PBS, fixed and immunostained for observation by confocal microscopy, as described below.

### *In vivo* effects of SVMPs on cremaster muscle vasculature (model with blood flow)

Groups of four mice were anesthetized with ketamine/xylazine and injected via the intrascrotal (i.s.) route with either 60 μg of BaP1 or 30 μg of CsH1, dissolved in 300 μl of PBS. These doses correspond to twice the dose used in the *ex vivo* model since each mouse has two cremaster muscles. PBS (300 μl) was used as control. Fifteen min after i.s. injection, mice were sacrificed by cervical dislocation and the cremaster muscles were dissected out, washed three times with PBS, fixed, and immunostained for observation by confocal microscopy, as described below.

### Immunostaining of mouse cremaster muscles

Tissues were fixed with 4% paraformaldehyde in PBS (for collagen IV and nidogen immunostaining) or methanol (for laminin immunostaining) for 30 min at 4°C. Whole tissues were incubated for 4 h at room temperature in blocking and permeabilization solution (12.5% goat serum, 12.5% fetal bovine serum, 0.5% Triton X-100 in PBS) under stirring at room temperature. Then, the tissues were incubated for 48 h at 4°C with either rabbit anti-collagen type IV polyclonal antibody (Abcam ab19808) at a dilution of 1:100, rabbit anti-nidogen 1 polyclonal antibody (Abcam ab14511) at a dilution of 1:200, or rabbit anti-laminin polyclonal antibody (Abcam ab11575) at a dilution of 1:100, to visualize the vascular BM. Simultaneously, the tissues were incubated with Cy3-labeled mouse anti-actin α smooth muscle monoclonal antibody (clone 1A4, Sigma C6198), at a dilution of 1:200, and anti-mouse vascular endothelial (VE) cadherin monoclonal antibody (clone BV14, eBioscience 14–1442), at a dilution of 1:200, to visualize the smooth muscle/pericytes and endothelial cell-to-cell junctions, respectively. The anti-VE cadherin was previously labeled with Alexa 647 according to the Alexa Fluor 647^®^ Monoclonal Antibody Labeling Kit (Molecular Probes A20186). After 48 h of incubation, tissues were washed with PBS and incubated for 4 h at 4°C with goat anti-rabbit polyclonal antibody (Invitrogen A11034). All the antibodies were diluted in PBS containing 10% fetal bovine serum. After 4 h of incubation, tissues were washed with PBS and the whole tissues were mounted on glass slides in PBS for confocal microscopy observation, as described in the next section.

### Analysis of tissues by confocal microscopy

Immunostained tissues were visualized using a Zeiss LSM 5 Pascal laser-scanning confocal microscope (Carl Zeiss Ltd) incorporating a 40X water objective (numerical aperture 0.8), and an Olympus Fluoview FV1000 laser-scanning confocal microscope incorporating a 40X oil objective (numerical aperture 1.3). Three-dimensional images of post-capillary venules (PCV), arterioles and capillaries in the tissue were acquired with sequential scanning of different channels at a resolution of 1,024 × 1,024 dpi, corresponding to a voxel size of 0.22 x 0.22 x 1.18 μm and 0.31 x 0.31 x 0.57 μm in the X × Y × Z plans, with the Zeiss and Olympus confocal microscope, respectively. Images of treated tissues were taken using the same settings used for control tissues for each experiment. At least five images of PCV and arterioles (20–45 μm diameter and 200 μm length) were obtained from the middle along the longitudinal axis until the lateral top segment of the vessel (i.e. semi-vessel) per tissue, whereas at least eight images of whole capillaries (200 μm length) were collected per tissue. Analysis of total fluorescence intensity (average gray value of all the pixels in the analyzed area), and size and density of gaps between adjacent smooth muscle/pericytes were carried out using Image J software. Three-dimensional reconstitution of the images was carried out using IMARIS x64 7.4.2 software.

### *In vivo* vascular permeability assay

To evaluate vascular permeability induced by SVMPs in the skin of mice, Evans Blue dye was used as marker of extravasation according to the protocol described by Radu and Chernoff [24], with some modifications. Briefly, groups of five animals (18–20 g) were injected by intravenous (i.v.) route in the lateral tail vein with 200 μl of Evans Blue dye (6 mg/ml), dissolved in PBS. After 5 min, mice were injected by intradermal (i.d.) route in the ventral abdominal region with either 2 μg of BaP1 or 1 μg of CsH1, dissolved in 50 μl of PBS. These doses were selected since they do not induce haemorrhage in the skin, as to avoid the development of a haemorrhagic lesion that would interfere with the measurement of Evans Blue extravasation, and in order to maintain the same protein mass ratio (2:1) used in the *in vivo* model described above. Controls received 50 μl of PBS alone by i.d. route. Fifteen minutes after i.d. administration, mice were sacrificed by cervical dislocation, their skin was removed, and the area of plasma, i.e. Evans Blue, extravasation was measured.

### Statistical analysis

Results were expressed as the mean ± standard error of mean (SEM). Statistical significance was determined by one-way analysis of variance (ANOVA) and multifactorial ANOVA (when more than one factor was analyzed) with Tukey as *post hoc* test using the IBM SPSS Statistics 22.0 and GraphPad Prim 6 software.

## Results

### Effects on BM components

**Fig 1:**
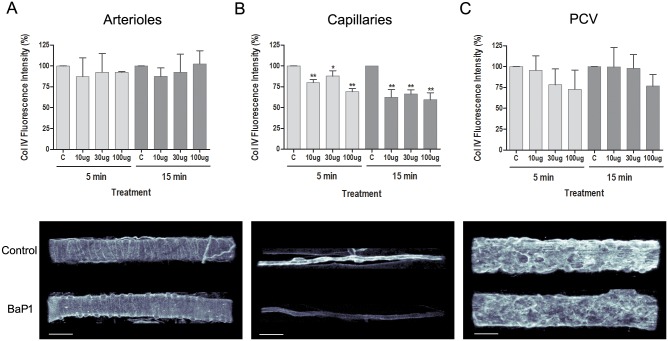
Dose and time dependency of BaP1 effects on type IV collagen from vascular BM on isolated mouse cremaster muscle. Isolated cremaster muscles were incubated with different amounts of BaP1 (10, 30 and 100 μg) for either 5 or 15 min (model without blood flow). Control tissues were incubated with PBS. Whole tissues were fixed and immunostained for observation by confocal microscopy and analysis of total fluorescence intensity for type IV collagen. Results are expressed as the mean ± SEM of the percentage of intensity related to control of at least five images of each vessel type: (A) arterioles, (B) capillaries, and (C) PCV per cremaster (n = 4). Below each graph, representative three-dimensional images of each vessel type immunostained for type IV collagen are shown with a gray color coding spectrum (black as low fluorescence intensity regions and white as high fluorescence intensity regions) for BaP1 (30 μg) and control at 15 min. The images show a decrease in fluorescence intensity for type IV collagen in BM of capillaries of treated tissues as compared to control, whereas no significant reduction was observed in arterioles and PCV. Scale bar represents 30 μm. *p < 0.05, **p < 0.001 as compared to control. C: control; Col IV: type IV collagen; PCV: post-capillary venules.

In order to study the dose and time dependence of the effects induced by BaP1 on the vasculature of cremaster muscle, and to define the optimal conditions for further studies, isolated tissues were incubated with different amounts of BaP1 for either 5 or 15 min. In conditions without blood flow and topical application of the toxin, BaP1 induced a decrease in fluorescence intensity of type IV collagen immunostaining of the BM of capillaries, as compared to controls, at multiple doses and two time points (Fig 1B). No significant differences were observed in the fluorescence intensity of type IV collagen of arterioles (Fig 1A) and PCV (Fig 1C); however, there was a trend to a dose-dependent decrease of the intensity in PCV.

**Fig 2:**
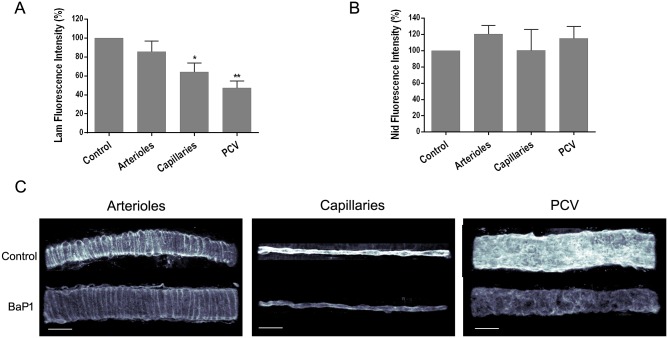
Effect of BaP1 on laminin and nidogen from vascular BM on isolated mouse cremaster muscle. Isolated cremaster muscles were incubated with 30 μg of BaP1 for 15 min (model without blood flow). Control tissues were incubated with PBS. Whole tissues were fixed and immunostained for observation by confocal microscopy and analysis of total fluorescence intensity for (A) laminin and (B) nidogen. Results are expressed as the mean ± SEM of the percentage of intensity related to control of at least five images of each vessel type per cremaster (n = 4). (C) Representative three-dimensional images of each vessel type immunostained for laminin are shown with a gray color coding spectrum (black as low fluorescence intensity regions and white as high fluorescence intensity regions). The images show a decrease in fluorescence intensity for laminin in BM of capillaries and PCV of treated tissues as compared to control, whereas no reduction in the fluorescence intensity was observed for nidogen. Scale bar represents 30 μm. *p < 0.05, **p < 0.001 as compared to control. Lam: laminin; Nid: nidogen; PCV: post-capillary venules.

The intermediate dose (i.e. 30 μg) and the time lapse of 15 min were selected to study the effect of BaP1 on other BM components in the *ex vivo* model. Under these conditions, BaP1 induced a decrease in fluorescence intensity for laminin at the BM of capillaries (p < 0.05) and PCV (p < 0.01), but not in arterioles (Fig 2A and 2C). Conversely, no significant differences in fluorescence intensity were observed in vascular BM immunostained for nidogen (Fig 2B).

**Fig 3:**
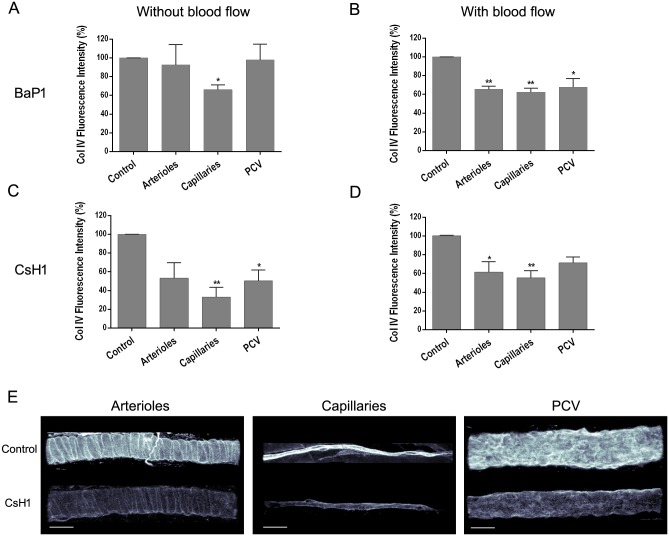
Effect of BaP1 and CsH1 on type IV collagen from vascular BM on mouse cremaster muscle. Isolated cremaster muscles were incubated with either (A) 30 μg of BaP1 or (C) 15 μg of CsH1 (model without blood flow). In another experiment, anesthetized mice were injected by intrascrotal route with either (B) 60 μg of BaP1 or (D) 30 μg of CsH1 (model with blood flow). Controls were incubated or injected with PBS. After 15 min of exposition to toxin in each model, whole cremaster muscles were fixed and immunostained for observation by confocal microscopy and analysis of total fluorescence intensity for type IV collagen. Results are expressed as the mean ± SEM of the percentage of intensity related to control of at least five images of each vessel type per animal (n = 4). (E) Representative three-dimensional images of each vessel type immunostained for type IV collagen are shown with a gray color coding spectrum (black as low fluorescence intensity regions and white as high fluorescence intensity regions) for CsH1 30 μg and control applied in anesthetized mice (i.e. model with blood flow). The images show a decrease in fluorescence intensity for type IV collagen in BM of arterioles, capillaries, and PCV of treated tissues as compared to control. Scale bar represents 30 μm. *p < 0.05, **p < 0.001 as compared to control. Col IV: type IV collagen; PCV: post-capillary venules.

In order to study the effects of BaP1 on vascular BM in conditions of blood flow, this SVMP was injected via the i.s. route to anesthetized animals. After 15 min, the tissues were dissected out for analysis. Type IV collagen was selected for these studies since it plays an important structural role in the mechanical stability of BM [25–30], and has been identified as a potential key target in the action of haemorrhagic SVMPs [6,7,17,31]. In contrast to the effect induced by BaP1 for BM type IV collagen in the *ex vivo* model, which occurred predominantly in capillaries (Fig 3A), this SVMP induced a widespread reduction in immunostaining in arterioles and PCV in the *in vivo* model, i.e. in conditions in which blood flow was present (Fig 3B).

In contrast to BaP1, the PIII SVMP CsH1 induced a decrease in fluorescence intensity for type IV collagen immunostaining in the three vessels types in the isolated tissue, i.e. without blood flow (Fig 3C), as well as in conditions in which flow was present (Figs 3D and 2E). The overall multivariate analysis of variance for the effect of BaP1 and CsH1 in the *ex vivo* and *in vivo* models highlights a difference between treatments (p < 0.001) and vessel types (p < 0.05), but not between presence or absence of blood flow.

### Effects on endothelial cell-to-cell junctions

**Fig 4:**
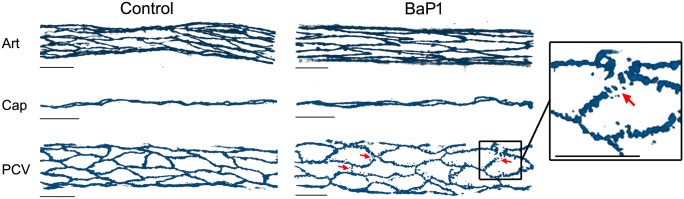
*In vivo* effect of BaP1 on endothelial cell-to-cell junctions on mouse cremaster muscle vasculature. Anesthetized mice were injected by intrascrotal route with 60 μg of BaP1 (model with blood flow). PBS was injected in controls. After 15 min, cremaster muscles were dissected out, fixed and immunostained for observation by confocal microscopy and analysis of the endothelial cell-to-cell junctions. Figure shows representative three-dimensional images from at least five images of each vessel type per animal (n = 4) immunostained for VE-cadherin. Notice the loss of junctional VE cadherin staining in PCV of treated tissues (arrows). Scale bar represents 30 μm. Art: arterioles; Cap: capillaries; PCV: post-capillary venules.

Antibodies against VE cadherin were used as markers to evaluate the effects of BaP1 and CsH1 on the endothelial cell-to-cell junctions of blood vessels. When SVMPs were evaluated *ex vivo* on the isolated mouse cremaster muscle, no significant changes were observed in total fluorescence intensity for VE cadherin staining or vascular endothelial morphology, as compared to controls. However, when effects were evaluated *in vivo*, a disruption in the alignment of VE-cadherin staining was observed in 50% of the analyzed images of PCV in tissues treated with BaP1, as compared to controls (Fig 4). In spite of this loss of continuous staining induced by BaP1, no significant decrease in total fluorescence intensity for VE cadherin staining was found in PCV in the *in vivo* model. This change in morphology was neither observed in arterioles nor capillaries after treatment with BaP1. In contrast, CsH1 did not induce any evident change in vascular endothelial morphology in any of the studied models.

### Effects on vascular permeability

**Fig 5:**
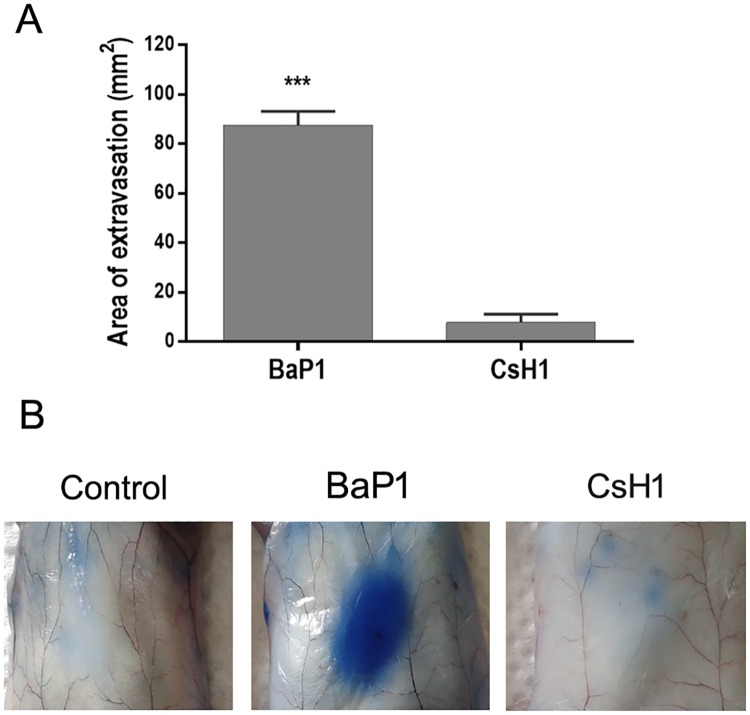
Effect of BaP1 and CsH1 on vascular permeability after intradermical application. Mice received an intravenous injection of 200 μl of Evans Blue dye (6 mg/ml). After 5 min, mice were injected by intradermal route, in the ventral abdominal region, with either 2 μg of BaP1 or 1 μg of CsH1. PBS was injected as control in another group of animals. After 15 min mice were sacrificed by cervical dislocation, their skin was removed and the area of extravasation was measured. (A) Results are expressed as the mean ± SEM (n = 5). **p < 0.001 as compared to CsH1. (B) Figure shows representative images from five animals analyzed.

In order to evaluate vascular permeability induced by both SVMPs, the area of plasma extravasation induced by toxin after i.d. administration was measured using Evans Blue dye as marker. The same protein mass ratio (2:1) used in the immunohistochemistry experiments was employed. An increase of vascular permeability was evident after administration of 2 μg of BaP1 with a mean extravasation area of 87 ± 13 mm^2^ (Fig 5). By contrast, 1 μg of CsH1 did not induce an increment in vascular permeability, since most of the tissues looked similar to controls, with only small areas of extravasation observed in some animals. Control animals did not show areas of extravasation in the skin.

### Effects on gaps between adjacent smooth muscle cells and pericytes

**Fig 6:**
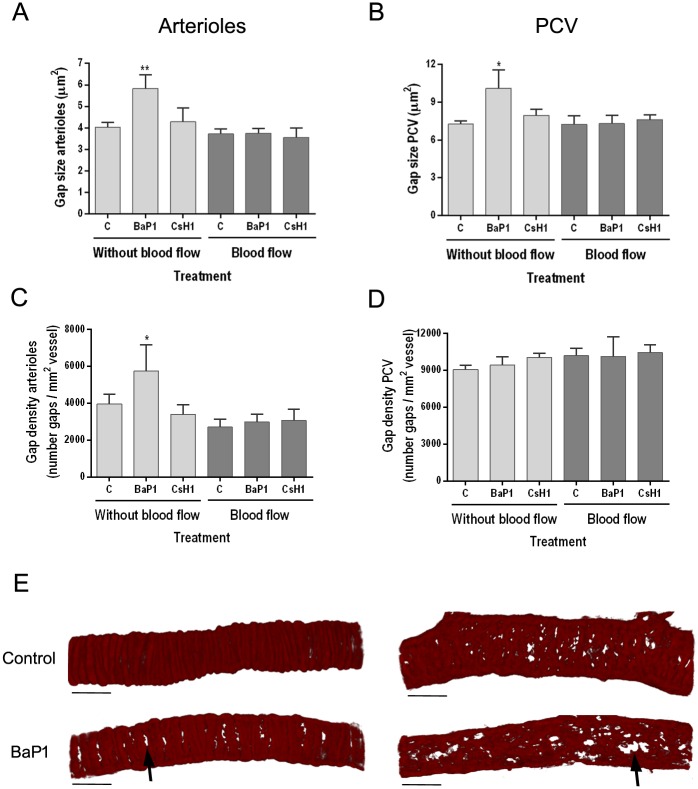
Effect of BaP1 and CsH1 on size and density of gaps between adjacent smooth muscle and pericytes on mouse cremaster muscle vasculature. Isolated cremaster muscles were incubated with either 30 μg of BaP1 or 15 μg of CsH1 (model without blood flow). In another experiment, anesthetized mice were injected by intrascrotal route with either 60 μg of BaP1 or 30 μg of CsH1 (model with blood flow). Controls were incubated or injected with PBS. After 15 min of exposition to toxin in each model, whole cremaster muscles were fixed and immunostained for observation by confocal microscopy and analysis of the gaps between adjacent smooth muscle and pericytes. Results are expressed as the mean ± SEM of the (A, B) gap size and (C, D) gap density (number of gaps per vessel area) of at least five images of arterioles and PCV per cremaster (n = 4). (E) Representative three-dimensional images of each vessel type immunostained for actin α smooth muscle are shown for BaP1 30 μg and control in the model without blood flow. Notice the increase in the gap size (arrows) in arterioles and PCV of treated tissues as compared to control. Scale bar represents 30 μm. *p < 0.05, **p < 0.001 as compared to control. C: control; PCV: post-capillary venules.

When BaP1 was applied directly on isolated mouse cremaster muscles (model without blood flow), a significant increase on the size of gaps between adjacent smooth muscle cells and pericytes was observed in arterioles (p < 0.001, Fig 6A) and PCV (p < 0.05, Fig 6B), as compared to controls. Moreover, this increment in gap size was observed together with an increase in gap density in arterioles (p < 0.05, Fig 6C), but not in PCV (Fig 6D). Conversely, when BaP1 was administered in the anesthetized animal (model with blood flow), no changes were observed in gap size and density for neither arterioles nor PCV. Furthermore, CsH1 did not induce any change in size and density of gaps in neither arterioles nor PCV in any of the studied models. The overall multivariate analysis of variance for the effect of BaP1 and CsH1 in the *ex vivo* and *in vivo* models indicated a difference between treatments (p < 0.01) and between conditions of presence and absence of blood flow (p < 0.01) for both arterioles and PCV.

## Discussion

This study assessed the action of two hemorrhagic SVMPs on the three main components of blood vessels in the microvasculature. Our observations clearly underscore that these SVMPs alter BM components in microvessels, particularly in capillaries. Previous studies have demonstrated the ability of these and other SVMPs to degrade type IV collagen, laminin and nidogen in different *in vivo* models [7,16–18,32]. However, since most of these studies used tissue homogenates and wound exudates, the precise origin of the degradation products detected is unknown. Our work demonstrates a decrease in the immunostaining of type IV collagen and laminin from the BM of blood vessels in a whole tissue preparation, as detected by confocal microscopy, suggesting a degradation of BM components by these enzymes.

The action of SVMPs on the vascular BM depends on their ability to reach this extracellular matrix structure, and to bind and degrade specific BM components. Moreover, the diffusion of degraded proteins and fragments away from the BM after hydrolysis also influences the immunostaining of these components in tissue preparations. Previous studies have demonstrated that PIII SVMPs co-localize with BM components of blood vessels to a higher extent than PI SVMPs, which have a more widespread distribution in the tissue [6,7]. This higher binding capacity of PIII SVMPs to microvessels is associated with the presence of exosites located in the Dis-like and Cys-rich domains of the toxin [6,33–37]. This could explain the greater effect of PIII SVMPs as compared to PI SVMP on the vascular BM on isolated tissue.

According to our results, both PI and PIII SVMPs degrade type IV collagen from the BM in capillaries to a higher extent than in PCV and arterioles. Moreover, it seems that the BM of PCV is more susceptible to degradation by SVMPs than its arteriolar counterpart. This might suggest that the ability of SVMPs to reach BM of PCV and arterioles is limited due to structural constraints in the wall of these vessels. However, BaP1 was able to degrade laminin in PCV, evidencing its ability to reach the BM of this vessel type. Moreover, it has been demonstrated that CsH1 binds to BM components of arterioles, capillaries, and PCV to a similar extent [7]. Taken together, these observations argue against the poor accessibility as the main cause behind the differences in the patterns of degradation of BM in various microvessel types.

The ability of BaP1 to reduce the immunostaining of laminin to a greater extent than type IV collagen might be due to the fact that type IV collagen constitutes a more stable covalently-linked network, in contrast to laminin [25–30]. Hence, it is likely that, even if type IV collagen is hydrolyzed, epitopes could remain within the BM, whereas laminin degradation products might be easily washed out from this structure. Previous investigations demonstrated the degradation of nidogen by SVMPs on tissue homogenates and on the BM preparation Matrigel *in vitro* [7,16,17,32]. Conversely, we did not observe a reduction in the immunostaining of nidogen, which may indicate that BaP1 does not degrade nidogen of vascular BM on the isolated cremaster muscle. One explanation for this disagreement is that epitopes could remain bound to BM even after nidogen is hydrolyzed by BaP1, which would be evidenced by western blot of tissue homogenates but not by immunohistochemistry. Another possible explanation is that nidogen and its degradation products detected in western blot of tissue homogenates might come from the BM of other tissue structures such as muscle and nerves.

When toxins were evaluated in the *in vivo* model, i.e. in the presence of blood flow, the effect of CsH1 on type IV collagen of blood vessels was similar to the effect seen in the *ex vivo* model. However, in the case of BaP1, loss of immunostaining was also evident in the BM of arterioles and PCV in the presence of blood flow, but not in its absence. This discrepancy is likely due to the different distribution of toxins in the tissue in these two experimental settings. In the *ex vivo* model, toxins were topically applied on the isolated tissue, i.e. in absence of blood flow. Instead, in the *in vivo* model SVMPs were applied by the i.s. route, in the presence of blood flow. Thus, the toxin distribution is likely to be different in these experimental models. Differences in temperature might also affect the distribution and enzymatic activity of the SVMPs, since the *ex vivo* model is performed at room temperature, whereas *in vivo* the toxins are acting at body temperature.

On the other hand, it has been described that the mechanical properties of the BM vary depending on the action of hemodynamic biophysical forces (i.e. wall tension and shear stress) operating under blood flow conditions in the microvasculature [38]. Therefore, under blood flow the susceptibility of BM components to the action of proteinases may be higher owing to the increased wall tension and mechanical stress. Studies have demonstrated that wall tension may play role in increasing the expression and activity of matrix metalloproteinases (MMPs) [39–41]. This hypothesis as related to SVMPs deserves further investigation. In addition, degradation products generated as a result of hydrolysis by SVMPs might be easily removed from the tissue *in vivo* whereby draining through the lymphatic vessels operate.

The degradation of type IV collagen in BM of capillaries induced by both SVMPs, with or without blood flow, supports the hypothesis that hydrolysis of this protein is a key event in the microvascular damage and haemorrhagic action of SVMP, as previously proposed [6,7,17,31]. Moreover, the fact that SVMPs reduce the immunostaining of type IV collagen in conditions of lack of blood flow lends support to the ‘two-step’ hypothesis for explaining the mechanism of action of hemorrhagic SVMPs [8]. The first step, i.e. hydrolysis of BM components, which can occur in the absence of flow as shown here, is a separate event from the second step, i.e. the distention and disruption of capillary wall integrity, which depends on blood flow.

Endothelial cells are an important component of vascular vessels. Previous work has shown alterations in vascular endothelial cells induced by haemorrhagic SVMPs, an effect associated *in vivo* with the distention of the capillary wall as a consequence of the hydrolysis and posterior weakening of the BM. This rapid effect *in vivo*, occurring within few min, is not due to a direct cytotoxic effect on endothelial cells [19,21,42–44]. It was therefore of interest to assess the action of SVMPs on endothelial cells in our model.

VE-cadherin is a transmembrane protein exclusively expressed by endothelial cells with an adhesive function in the vascular cell-cell contact. VE cadherin plays an important role in the microvascular integrity [45,46] and contributes to the regulation of vascular permeability [47]. Our results demonstrate that BaP1 is able to disrupt the endothelial barrier in PCV in the *in vivo* model. This change in the morphology induced by BaP1 in cell-cell junctions is similar to that described after an inflammatory stimulus, and is associated with changes in VE-cadherin localization, internalization or disassembly [47–49]. Interestingly, a mechanism of SVMP-induced extravasation, known as haemorrhage *per diapedesis*, has been described in PCV; in this case erythrocyte extravasation occurs through widened intercellular junctions in venular endothelial cells [11]. Thus, the possible effect of SVMPs in endothelial cells junctions in PCV, using VE-cadherin as a marker, may provide clues on the mechanism of haemorrhage *per diapedesis*.

The observed effect of BaP1 on VE-cadherin could be due to proteolysis. However, the fact that such effect occurred in the *in vivo* model, but not in the *ex vivo* setting, and that VE-cadherin would be accessible for hydrolysis in both experimental conditions, argues against this explanation. On the other hand, it was somehow surprising that CsH1 did not exert this effect of VE-cadherin. An explanation for this apparently puzzling observation may have to do with the different pro-inflammatory activity of these toxins, evidenced by their ability to induce extravasation of Evans Blue. BaP1 exerts a strong pro-inflammatory action, causing oedema associated with the synthesis and release of several inflammatory mediators [21,22,50–53]. In contrast, CsH1 exerts a much lower increase in plasma extravasation in our experimental setting at the dose tested. Since increments in vascular permeability are associated with opening of endothelial cell junctions, which in turn involve VE-cadherin, the different effect described for the action of these two SVMPs in this cell marker can be explained on the basis of their different pro-inflammatory activity. This mechanism would operate *in vivo* but not in *ex vivo* conditions where blood flow is absent.

Other important components of the microvasculature are smooth muscle cells and pericytes. In most tissues arterioles are surrounded by layers of smooth muscle cells with tight junctions between adjacent cells, whereas PCV are irregularly covered by smooth muscle cells, and have a net-like cell layer of pericytes embedded within the venular BM, with gaps between adjacent cells [54–56]. The effects of SVMPs on these vascular components have not been studied before and may, therefore, illustrate a hitherto unknown aspect of SVMP-induced local pathology.

Our results demonstrate that BaP1 induces an increase in density of gaps between smooth muscle cells/pericytes in arterioles but not in PCV in *ex vivo* conditions, i.e. without blood flow. Thus, new gaps are created in arterioles, while there is an increase in the size of pre-existing gaps in PCV. On the other hand, in the presence of blood flow these effects on arterioles and PCV were not observed for either toxin.

This increase in gap size in PCV and neo-formation of gaps in arterioles could be associated with cell damage or cell contraction. Previous studies have described an increase in the gap size in PCV after inflammatory stimulus [57–59]. However, when BaP1 was injected in the tissue with blood flow, in conditions where inflammation develops, no effects on gaps were observed, thus arguing against inflammation as the cause of these phenomena. Interestingly, CsH1 did not induce any effect on either gap size and gap density. This could be explained on the basis of the presence of exosites in the extra domains of CsH1, which are likely to direct and concentrate this SVMP in the BM of the vessels. In contrast, BaP1 can operate on different tissues or structures due to the lack of targeting of this SVMP to vessel walls, as described previously [7]. Thus, the different ‘directionality’ of SVMPs having variable domain structure may impact on their ability to damage capillaries, arterioles and PCV.

Pericytes are closely associated with endothelial cells, and play a role in maintaining the integrity of the vessel walls, vessel contractility, regulation of blood flow, vascular BM remodeling during inflammation, vascular development, angiogenesis, and wound healing [59–62]. Thus, the effects of SVMPs on arteriolar smooth muscle cells and pericytes could play an important role in the pathogenesis of tissue damage and constitute an unexplored target for the search of novel therapeutic avenues in snakebite envenoming. Previous studies have demonstrated that *B*. *asper* venom affects the smooth muscle of lymphatic vessels through the action of myotoxic phospholipases A_2_ [63]; however, our findings on the action of SVMPs on smooth muscle cells and pericytes have not been previously described for a SVMP. In the context of the overall pathogenesis of viperid venom-induced local tissue damage, it is likely that arteriolar and other smooth muscle cells might be affected by the combined action of myotoxic phospholipases A_2_ and SVMPs, especially PI SVMP.

## Conclusions

The study of the *ex vivo* and *in vivo* effects of SVMPs on whole tissue using high resolution confocal microscopy techniques provides new insights into the effects of SVMPMs on the three components of microvasculature. Our findings demonstrate that both haemorrhagic SVMPs are able to degrade type IV collagen in BM of capillaries *in vivo*, which supports the hypothesis that hydrolysis of this protein is an important event in the haemorrhagic action of these toxins. Moreover, BaP1 disrupts the endothelial barrier in PCV and increases vascular permeability. Furthermore, BaP1 is able to create new gaps between smooth muscle cells in arterioles and increase gap size between pericytes in PCV in *ex vivo* conditions. In contrast, the action of the PIII SVMP CsH1 is more directed towards the BM of microvessels, probably as a consequence of the presence of exosites in various domains of this enzyme, which direct it to targets in the BM. Our results underscore the complexity of the pathological effects induced by SVMPs in the microvasculature.
